# Effect of probiotic interventions on oral health in children: a systematic review

**DOI:** 10.3389/fdmed.2026.1914359

**Published:** 2026-09-16

**Authors:** Smruti Bondre, Vabitha Shetty, Mausam Kumari, Urvashi Shetty

**Affiliations:** 1 Department of Pediatric and Preventive Dentistry, AB Shetty Memorial Institute of Dental Sciences, NITTE (Deemed to be University), Mangalore, Karnataka, India; 2 Department of Oral and Maxillofacial Pathology and Microbiology, AB Shetty Memorial Institute of Dental Sciences, NITTE (Deemed to be University), Mangalore, Karnataka, India

**Keywords:** early childhood caries, oral microbiome, paraprobiotics, preschool children, probiotics, *Streptococcus mutans*, synbiotics, systematic review

## Abstract

**Introduction:**

Early childhood caries (ECC) is one of the most prevalent chronic diseases affecting young children worldwide. Probiotic supplementation has emerged as a potential adjunctive strategy for modifying the oral microbiome and reducing caries risk; however, evidence regarding its effectiveness remains inconsistent. This review evaluated the effects of probiotic interventions on oral health outcomes in children under 6 years of age, with emphasis on probiotic-based interventions and their influence on caries incidence, microbial changes, and biological mechanisms.

**Methods:**

A systematic search of PubMed, Scopus, and Web of Science was conducted in accordance with the PRISMA 2020 guidelines up to June 2026. Eligible studies included randomized controlled trials, non-randomized intervention studies, and *in vitro* mechanistic studies evaluating probiotic interventions in children aged 0–6 years. Two independent reviewers performed study selection, data extraction, and quality assessment using the Cochrane RoB 2.0, ROBINS-I, and QUIN tools. Due to substantial clinical and methodological heterogeneity, findings were synthesized narratively, with supplementary quantitative analyses performed where appropriate. Certainty of evidence was assessed using the GRADE framework.

**Results:**

Twenty-one studies met the inclusion criteria, comprising 13 randomized controlled trials, three non-randomized intervention studies, and five *in vitro* studies. The clinical evidence primarily evaluated probiotic supplementation, most commonly *Lactobacillus paracasei* SD1, *Lactobacillus rhamnosus* strains, and *Limosilactobacillus reuteri*. Most randomized trials reported reductions in caries incidence or progression and decreases in salivary *Streptococcus mutans* counts, although findings were heterogeneous across probiotic strains and outcome measures. Mechanistic studies consistently demonstrated antimicrobial and antibiofilm activity of probiotic lactobacilli against cariogenic microorganisms, providing biological plausibility for the observed clinical effects. GRADE assessment indicated moderate-certainty evidence for randomized controlled trials, while evidence from non-randomized and *in vitro* studies was rated as low and very low certainty, respectively.

**Conclusion:**

Probiotic supplementation appears to be a promising adjunct to conventional preventive strategies for improving oral microbial profiles and reducing caries progression in preschool children. However, the observed benefits appear to be strain-specific, and considerable heterogeneity across interventions and outcome measures limits definitive clinical recommendations. Further high-quality, adequately powered randomized controlled trials with standardized probiotic regimens, uniform caries assessment.

**Systematic Review Registration:**

https://www.crd.york.ac.uk/PROSPERO/view/CRD420251229276, PROSPERO CRD420251229276.

## Introduction

Early childhood caries (ECC) is a significant global public health concern that disproportionately affects children from low socioeconomic backgrounds and marginalized communities (1). ECC is multifactorial, arising from interactions among dietary habits, oral hygiene practices, fluoride exposure, host susceptibility, and the ecological balance of the oral microbiome (2). Emerging evidence underscores the interconnected nature of the oral and gut microbiomes, including a shared early colonization route through the milk–oral–gut axis, which influences microbial maturation, immune development, and caries susceptibility during early life (3, 4). Imbalances in the gut microbiome have shown a significant influence on the development of oral disease (5). Distinctive oral and gut microbiome profiles have been identified in children with caries and extrinsic black stains, suggesting systemic microbial involvement beyond localized oral dysbiosis (6).

These changes in gut microbiota can be addressed with the usage of aids such as probiotics, synbiotics, and other dietary interventions to induce modulation of the gut microbiome, and in turn the oral microbiome. Hegde et al. identified increased bioavailability of salivary calcium and phosphate levels with the administration of milk, cheese, and GC tooth mousse among children as a preventive measure (7). Jamil conducted a systematic review and highlighted the positive impact of dietary interventions on different aspects of oral health, such as periodontal disease and development of dental caries (8). Meng et al. evaluated the efficacy of probiotics on oral health via a meta-analysis and reported that they could significantly reduce the *Streptococcus mutans* (*S. mutans*) count in saliva, especially with the probiotic *Lactobacillus rhamnosus* (9). The taxonomy of the genus *Lactobacillus* was revised in 2020, resulting in the reclassification of several species into new genera. Accordingly, updated nomenclature is used throughout this review [e.g., *Limosilactobacillus reuteri* (formerly *Lactobacillus reuteri*), *Ligilactobacillus salivarius* (formerly *Lactobacillus salivarius*), *Lacticaseibacillus rhamnosus* (formerly *Lactobacillus rhamnosus*), and *Lacticaseibacillus paracasei* (formerly *Lactobacillus paracasei*)].

The literature evaluating the impact of probiotic interventions on oral health is varied and does not provide a definitive consensus. A randomized clinical trial in 2022 evaluating the effect of *Limosilactobacillus reuteri* could not detect a significant change in the occurrence of ECC among preschool children (10). However, another probiotic strain (ProBiora3®) evaluated in 2015 demonstrated a promising effect on ECC prevention (11). Similarly, several probiotic strains have demonstrated potential benefits in preschool children, including Lactobacillus reuteri (10), Limosilactobacillus salivarius (live and inactivated) (12, 13), Lactobacillus rhamnosus SP1 delivered via milk (14), and the ProBiora3® blend consisting of Streptococcus uberis, S. oralis, and S. rattus (11). Mechanistic studies have reinforced their biological plausibility, demonstrating that probiotic lactobacilli can inhibit the growth, acidogenicity, and biofilm formation of Streptococcus mutans *in vitro* (15).

Additionally, dietary patterns, particularly the consumption of unsweetened milk, cheese, and yogurt, have been associated with a lower caries prevalence, whereas sweetened dairy products increase the caries risk in young children (16). Biochemical evidence supports the protective role of dairy-derived calcium and phosphate in enhancing remineralization (7).

Despite these promising findings, substantial heterogeneity persists across the literature, with variations in probiotic strains, dosage, delivery vehicles, follow-up durations, outcome measures (ICDAS vs. dmft), and risk of bias. Previous reviews have primarily focused on probiotics alone or have lacked integration of emerging para-probiotic trials and microbiome-focused observational studies that evaluate oral–gut interaction pathways (17). Therefore, this systematic review aimed to synthesize the current evidence on probiotic and other microbiome-modulating interventions and their effects on oral health outcomes in children under 6 years of age, with an emphasis on the caries incidence, microbial changes, and mechanistic pathways.

## Materials and methods

### Protocol and registration

This systematic review was conducted in accordance with the PRISMA 2020 guidelines. The protocol was prospectively registered with PROSPERO (2025 CRD420251229276).

### Eligibility criteria

Inclusion criteria:

Population:

- Children aged 0–6 years
- Healthy or ECC/SECC-diagnosed children.

Interventions:

- Ingested probiotics, paraprobiotics, synbiotics, fermented dairy products, and dietary components with microbiome-modulating effects.
- Evaluation of oral or gut microbiota in relation to dental caries.
- *In vitro* mechanistic studies relevant to the pediatric population.

Comparators:

- Placebo, standard care, no intervention, or comparison group.

Outcomes:

- Primary: caries incidence/increment (ICDAS Detection and dmft/dmfs).
- Secondary outcomes included microbial counts (*S. mutans* and lactobacilli), biofilm changes, saliva biomarkers, microbiome composition, plaque index, and gingival inflammation.

Study designs included:

- Randomized controlled trials (RCTs)
- Cluster RCTs
- Non-randomized clinical trials
- Cross-sectional observational studies
- *In-vitro* mechanistic studies (supportive evidence only)

Exclusion criteria:

- Studies involving children >6 years
- Reviews, letters, editorials
- Animal studies
- Lack of extractable outcome data
- Interventions delivered as lozenges or oral rinses

### Selection process

#### Literature screening and data extraction

Literature screening was performed independently by two reviewers using Rayyan (18). Reviewers were blinded to each other's screening decisions throughout the independent screening process to minimize selection bias. Any disagreements were resolved through discussion, with consultation from a third reviewer when necessary.

A two-stage screening process was applied (Figure 1).

1. Title and abstract screening
2. Full-text review

**Figure 1:**
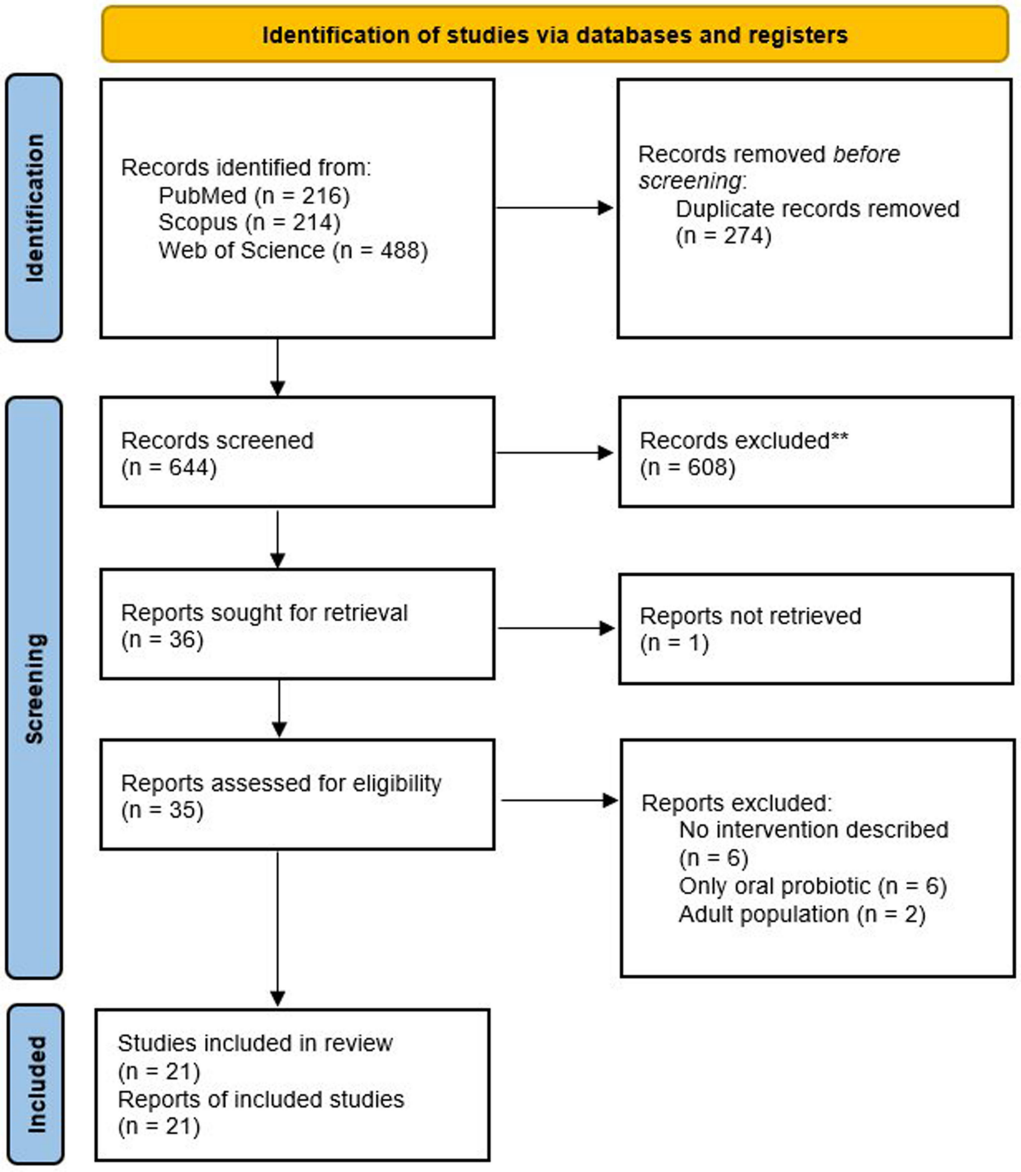
PRISMA flow diagram depicting literature search and selection.

Data extraction was performed using a pre-piloted, standardized form. The following data were extracted from each included study: Author, year, country, study design, participant characteristics (sample size, age, and baseline oral health status), intervention or exposure characteristics (type of probiotic/paraprobiotic or microbiome-modulating strategy, strain, dose, frequency, duration, and comparator), duration of follow-up, primary and secondary outcomes (caries incidence/increment, microbial counts, plaque and gingival indices, salivary biomarkers, microbiome composition), and the principal findings relevant to the review objectives (Table 1).

**Table 1:** Search strategy.

S.No	Database	Search strategy	Results
1	PubMed	(“Probiotics”[Mesh] OR “Prebiotics”[Mesh] OR synbiotic*[tiab] OR postbiotic*[tiab] OR probiotic*[tiab] OR prebiotic*[tiab] OR “Fermented Foods”[Mesh] OR fermented food*[tiab] OR “Dietary Fiber”[Mesh] OR fiber[tiab] OR fibre[tiab] OR “Gastrointestinal Microbiome”[Mesh] OR “gut microbiome”[tiab] OR “gut microbiota”[tiab] OR “intestinal microbiome”[tiab]) AND (“Dental Caries"[Mesh] OR “early childhood caries”[tiab] OR ECC[tiab] OR “oral microbiome”[tiab] OR “oral microbiota”[tiab] OR “streptococcus mutans”[tiab]) AND (“Infant”[Mesh] OR “Child, Preschool”[Mesh] OR infant*[tiab] OR toddler*[tiab] OR preschool*[tiab]) NOT (“Review”[Publication Type] OR “Systematic Review”[Publication Type] OR “Meta-Analysis”[Publication Type] OR animals[mh] NOT humans[mh] OR mouthwash*[tiab] OR “mouth rinse”[tiab] OR lozenge*[tiab] OR toothpaste[tiab] OR “oral probiotic”[tiab] OR “topical probiotic”[tiab])	216
2	Scopus	[TITLE-ABS-KEY (probiotic* OR prebiotic* OR synbiotic* OR postbiotic* OR “fermented food*” OR “dietary fiber” OR fiber OR fibre OR “gut microbiome” OR “gut microbiota” OR “intestinal microbiome”)] AND [TITLE-ABS-KEY (“dental caries” OR “early childhood caries” OR ECC OR “oral microbiome” OR “oral microbiota” OR “streptococcus mutans” OR “oral health” OR “oral hygiene” OR “dental plaque”)] AND [TITLE-ABS-KEY (infant* OR toddler* OR preschool* OR “preschool child*” OR “young child*”)] AND NOT [TITLE-ABS-KEY (mouthwash* OR lozenge* OR toothpaste OR “topical probiotic”)] AND [LIMIT-TO (LANGUAGE, “English”)] AND (EXCLUDE (DOCTYPE, “re”) OR EXCLUDE (DOCTYPE, “no”) OR EXCLUDE (DOCTYPE, “cp”) OR EXCLUDE (DOCTYPE, “ch”) OR EXCLUDE (DOCTYPE, “cr”) OR EXCLUDE (DOCTYPE, “le”) OR EXCLUDE (DOCTYPE, “ed”) OR EXCLUDE (DOCTYPE, “bk”))	214
3	Web of Science	[ALL (probiotic* OR prebiotic* OR synbiotic* OR postbiotic* OR “fermented food*” OR “dietary fiber” OR fiber OR fibre OR “gut microbiome” OR “gut microbiota” OR “intestinal microbiome”)] AND [ALL (“dental caries” OR “early childhood caries” OR ECC OR “oral microbiome” OR “oral microbiota” OR “streptococcus mutans” OR “oral health” OR “oral hygiene” OR “dental plaque”)] AND [ALL (infant* OR toddler* OR preschool* OR “preschool child*” OR “young child*”)]	488

#### Risk of bias

Risk of bias assessment for the included studies was conducted using the appropriate tools as per the study design. The Cochrane RoB 2.0 tool was used for randomized clinical trials (19). The Risk Of Bias In Non-Randomized Studies of Interventions (ROBINS-I) tool (20) was used for the non-randomized study designs. The Quality Assessment Tool for *in vitro* Studies (QUIN) (21) was used for evaluating the *in-vitro* study designs. The scoring was conducted as per the designated scores for each tool and the reporting guidelines were adhered to during the quality assessment process.

## Results

### Study selection

Database searching identified 918 records (PubMed: 216; Scopus: 214; Web of Science: 488). After removal of 274 duplicates, 644 unique records were screened at title and abstract level. Following title/abstract screening, 36 records were selected for full-text assessment. The reasons for exclusion from the full-text screening are depicted in Figure 1. A final set of 21 articles were included in the review of which 13 articles were randomized controlled trials, three were non-randomized interventional studies, and five were *in-vitro* studies. One study was composed of both *in-vitro* and cross-sectional datasets (29).

### Study characteristics

The studies conducted in the present review were spread globally, including Finland (22), Thailand (23–27), Sweden (10, 28), Egypt (29), Iran (30), Turkey (31), Colombia (32, 33), Poland (34), Chile (14, 35, 36), USA (37), China (15, 38), and India (39).

The clinical interventions were predominantly delivered via milk (14, 22, 28, 32, 35, 36). One study used milk tablets (23), two studies used yogurt (31, 38), and two studies used oral drops (10, 30). The probiotic strains used in the clinical interventions varied considerably, spanning *Lactobacillus rhamnosus* (*GG*, *LB21*, *SP1*, *ATCC 15820*), *Limosilactobacillus reuteri, Lactobacillus paracasei SD1, Bifidobacterium longum, and multi-strain yogurt cultures*, with follow-up durations ranging from 2 weeks (30) to 21 months (28) (Table 2).

**Table 2:** Characteristics of included clinical studies.

S.No.	Author (year)	Country	Study design	Population	Sample size (I/C)	Age (range)	Intervention	Comparator	Delivery vehicle	Duration/follow-up	Outcomes assessed	Key findings
1	Näse et al. (2001) (22)	Finland	Randomized controlled trial	Children	594	1–6 years	Lacticaseibacillus rhamnosus GG, ATCC 53,103, bacteria 5–10E105 CFU/mL)	Placebo	Milk	7 months	Caries risk, LGG colonization in salivary and fecal samples	LGG was found to reduce the risk of caries significantly. The effect was more prominent in 3–4 year olds.
2	Stecksén-Blicks et al. (2009) (28)	Sweden	Cluster randomized trial	Children	248	1–5 years	2.5 mg/L fluoride and 10 7 CFU/mL L. rhamnosus LB21	Placebo	Milk	21 months	Caries increment and salivary bacterial count	Daily consumption of milk containing probiotic bacteria and fluoride reduced caries in preschool children with a prevented fraction of 75%.
3	Rodriguez et al. (2016) (14)	Chile	Cluster randomized trial	Children	261	2–3 years	Lacticaseibacillus rhamnosus SP1 (107 CFU/mL)	Placebo	Milk	40 weeks	ICDAS caries progression	The regular long-term intake of probiotic-supplemented milk may reduce caries development in high-caries preschool children
4	Tehrani et al. (2016) (30)	Iran	Randomized controlled trial	Children	61	3–6 years	Lacticaseibacillus rhamnosus ATCC 15,820 [1 × 1,010 colony-forming unit (CFU)/mL], Limosilactobacillus reuteri ATCC 55,730 (2 × 109 CFU/mL), and Bifidobacterium longum subsp. infantis ATCC 15,697 (1.5 × 109 CFU/mL)	Placebo	Oral drops	2 weeks	Salivary S. mutans and Lactobacillus counts	Use of this probiotic drop decreased salivary counts of SM; however, LB counts did not change. In addition, use of the drop in children with higher salivary counts appeared to be more effective
5	Villavicencio et al. (2017) (32)	Colombia	Cluster randomized trial	Children	363	3–4 years	Lacticaseibacillus rhamnosus was 5 × 106 CFU/g and the Bifidobacterim longum 3 × 106 CFU/g	Placebo	Milk	9 months	ICDAS caries progression and salivary bacterial counts	Regular consumption of milk containing probiotics bacteria reduced CFU/mL of Lactobacillus spp. and increased salivary buffering capacity at 9 months of consumption.
6	Eden et al. (2018) (31)	Turkey	Case-control	Infant-mother pairs	27	6–8 months	probiotic yogurt (Bifidobacterium Longum BB536, Bifidobacterium Bifidum Bb12, Lacticaseibacillus rhamnosus HN001)	Home made yogurt	Yogurt	3 weeks	Salivary pH, buffering capacity, and Streptococcus mutans, Lactobacilli and sIgA levels	Salivary pH, IgA, S. mutans and lactobacilli values showed no significant change after 3 weeks of probiotic yogurt consumption, however there was a statistically significant increase in the buffering capacity of saliva.
7	Pahumunto et al. (2018) (25)	Thailand	Randomized controlled trial	Children	124	1.5–5 years	Lacticaseibacillus paracasei SD1 107 CFU/g	Placebo	Milk	6 months	Association between probiotic consumption and bacterial level, and caries progression	Consumption of milk powder containing Lacticaseibacillus paracasei SD1 resulted in a reduction of both salivary MS and delayed new caries development, and the strain is safe for use in young children.
8	Angarita-Díaz et al. (2019) (33)	Colombia	Quasi-experimental	Children	63	3–5 years	Lacticaseibacillus rhamnosus GG (7.5 × 105 CFU/mL of milk) and B. longum (4.5 × 105 CFU/mL of milk).	Milk powder	Milk	3 months	plaque index, salivary pH, pH variation before and after a sugar rinse, quantification of Streptococcus mutans in saliva and demineralisation of the carious lesions	Lactic acid probiotics can contribute to the decrease in the number of cariogenic microorganisms.
9	Manmontri et al. (2020) (26)	Thailand	Multi-center randomized controlled trial	Children	487	3–5 years	viable 1.8 × 107 CFU/mL of Lacticaseibacillus paracasei SD1	Placebo	Milk	12 months	Absolute quantities of S. mutans and total lactobacilli in the saliva and plaque samples assessed by qPCR, caries detection	Probiotic administration daily or triweekly reduces S. mutans quantities, whereas it increases total lactobacilli quantities that persists at least 6 months after discontinuation in the saliva and plaque samples of preschool children.
10	Piwat et al. (2020) (24)	Thailand	Multi-center randomized controlled trial	Children	487	1–5 years	107 colony-forming units (CFU)/g of viable Lacticaseibacillus paracasei SD1	Placebo	Milk	12 months	Caries transition, including caries progression and regression	probiotic milk consumption either daily or triweekly can modestly prevent new caries, but considerably reverse carious lesions, suggesting that a daily or triweekly dose interval is sufficient to reverse carious lesions
11	Sandoval et al. (2021) (36)	Chile	Randomized controlled trial	Children	42	2–3 years	107-CFU/mL Lacticaseibacillus rhamnosus SP1	Placebo	Milk	10 months	salivary concentration of human β-defensin-3 (hβD-3) and ICDAS scores	Regular intake of probiotic-supplemented milk in preschool children with high caries risk decreased the occurrence of caries and the salivary levels of hβD-3
12	Wattanarat et al. (2021) (27)	Thailand	Randomized controlled trial	Children	487	1–5 years	Lacticaseibacillus paracasei SD1 at 107 colony-forming units (CFU) per gram	Placebo	Milk	12 months	Streptococcus mutans and total lactobacilli levels using qPCR and for human neutrophil peptides 1–3 (HNP1–3) levels using ELISA	In the severe-ECC status, daily or triweekly consumption of Lacticaseibacillus paracasei SD1 significantly enhanced salivary HNP1–3 levels, but reduced S. mutans levels, possibly resulting in reduction of caries progression
13	Xu et al. (2021) (38)	China	Clinical trial	Children	30	4–6 years	Lactobacillus bulgaricus, Streptococcus thermophilus, Lactobacillus acidophilus, Bifidobacteria lactobacillus, and Lactobacillus casei	Placebo	Yogurt	12 months	Salivary microbial DNA extraction and profiling to assess changes in oral microbiota	The α diversity analysis showed that salivary microbial diversity and richness were similar between the groups. The β diversity analysis showed that salivary microbial community structure changed with the consumption of probiotic yogurt.
14	Elazim et al. (2022) (29)	Egypt	Cross-sectional and *in vitro*	Infants	118	2–6 months	Probiotic supplemented formula	Breast milk and plain infant formula	Milk	N/A	Salivary bacterial detection and bacterial colony counts	Lactobacilli were detected in all infants, while no significant differences were found in S. mutans detection among groups. Counts of both microorganisms in saliva were lowest in probiotic group
15	Hasslöf et al. (2022) (10)	Sweden	Randomized controlled trial	Children	38	2–5 years	Two strains of the probiotic bacteria Limosilactobacillus reuteri (DSM 17,938 and ATCC PTA 5289) with a minimum of 100 million live bacteria of each strain.	Placebo	Oral drops	12 months	Caries prevalence (decayed, extracted, and filled teeth/surfaces; deft/defs) from the dental records. Family characteristics, dietary habits, and oral hygiene routines collected via questionnaire.	High rate of recurrent moderate and extensive lesions after 12 months (67%) but there were no significant differences between the groups. There were no beneficial effects on dental plaque or gingival inflammation.
16	Ibacache et al. (2025) (35)	Chile	Randomized controlled trial	Children	205	2–3 years	Lacticaseibacillus rhamnosus SP1 probiotic	Placebo	Milk	12 months	ICDAS caries progression	Daily consumption of milk supplemented with Lacticaseibacillus rhamnosus SP1 for 10 months reduced the number of preschool children who developed cavitated carious lesions over a 12-month period and may represent a safe and feasible adjunct for community-based prevention of early childhood caries.
17	Wongsupa et al. (2023) (23)	Thailand	Cluster randomized trial	Children	260	2–5 years	Lacticaseibacillus rhamnosus SD11	Placebo	Milk tablets	12 months	dmfs scores and cost-effectiveness ratio (CER)	There were significantly fewer caries among study subjects in the intervention group than the control group but the intervention was costly

The population evaluated in the present review is composed of 3,895 children aged approximately 1–6 years. A total of 1,748 children received the active intervention and 1,746 received placebo or standard care across the RCT tier. The single observational study (31) compared probiotic yogurt to home-made yogurt in 27 infant–mother pairs over 3 weeks. The two non-randomized intervention studies (33, 38) together enrolled 69 children. The five *in-vitro* studies (15, 29, 34, 37, 39) used clinical *S. mutans*, *Lactobacillus*, and *Candida albicans* isolates or plaque-derived biofilms from pre-school children and assessed antimicrobial and antibiofilm effects of various Lactobacillus species using disc-diffusion, biofilm, and molecular assays.

### Quality assessment of the included studies

The Cochrane RoB 2.0 tool (19) was used for the 13 clinical trials. No trial was rated an overall high risk, whereas two trials were rated an overall low risk of bias (25, 30). The remaining eight assessed trials were provided an overall rating of some concern (10, 14, 22, 23, 28, 32, 35, 36). Three clinical trials (24, 26, 27), were not assessed separately as they were additional outcomes and subsets assessed as part of a larger clinical trial (25). The randomization-process domain accounted for the largest share of these downgrades (14, 22, 23, 28, 36), followed by missing outcome data (10, 22) and selection of the reported result (10, 36). Deviations from the intended interventions affected one trial (36). Notably, Villavicencio et al. (32) and Ibacache et al. (35) were rated low risk on all five displayed domains yet carry an overall some-concerns judgment—both are cluster-randomized trials, and this pattern is consistent with an unreported cluster-specific domain (e.g., recruitment bias, or timing of identification/recruitment of individual participants in relation to cluster randomization) driving the overall rating without appearing in the five-domain summary shown (Figure 2).

**Figure 2:**
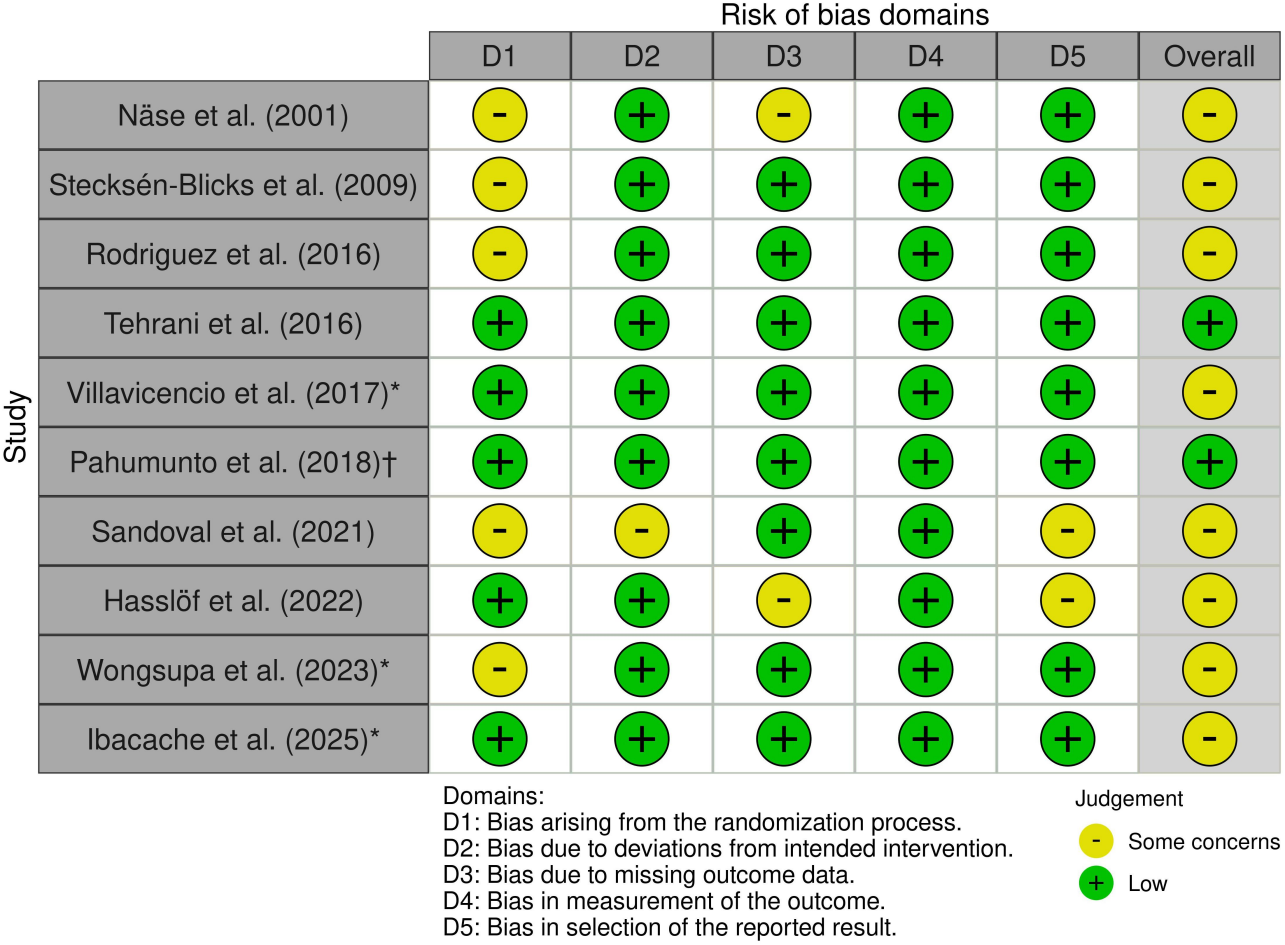
Risk of bias summary of the included clinical trials. For cluster-randomized trials [Villavicencio et al. (32), Wongsupa et al. (23), and Ibacache et al. (35)], the overall risk-of-bias judgment incorporated the additional RoB 2 cluster-specific domain (bias arising from the timing of identification and recruitment of participants), which is not displayed in the standard robvis visualization. ^†^Pahumunto et al. (25) represents the Thai multicentre randomized controlled trial, which was also reported by Manmontri et al. (26), Piwat et al. (24), and Wattanarat et al. (27). Risk of bias was assessed at the trial level.

### Risk of bias in non-randomized studies

The risk of bias for the non-randomized intervention studies were conducted using the ROBINS-I tool (20), assessed across seven domains. Two of the studies were scored moderate (31, 33) and one was scored serious (38) in the confounding domain. Selection of participants was moderately affected in one study (38) whereas deviation from the intended interventions were noted to be at greater risk in two studies (31, 38). An outcome of significant concern in all three studies was the reporting of missing data. The overall scoring was graded as “serious” for Eden et al. (31), “moderate” for Angarita-Diaz et al. (33), and “critical” for Xu et al. (38). The findings are reported in a traffic light matrix plot (Figure 3).

**Figure 3:**
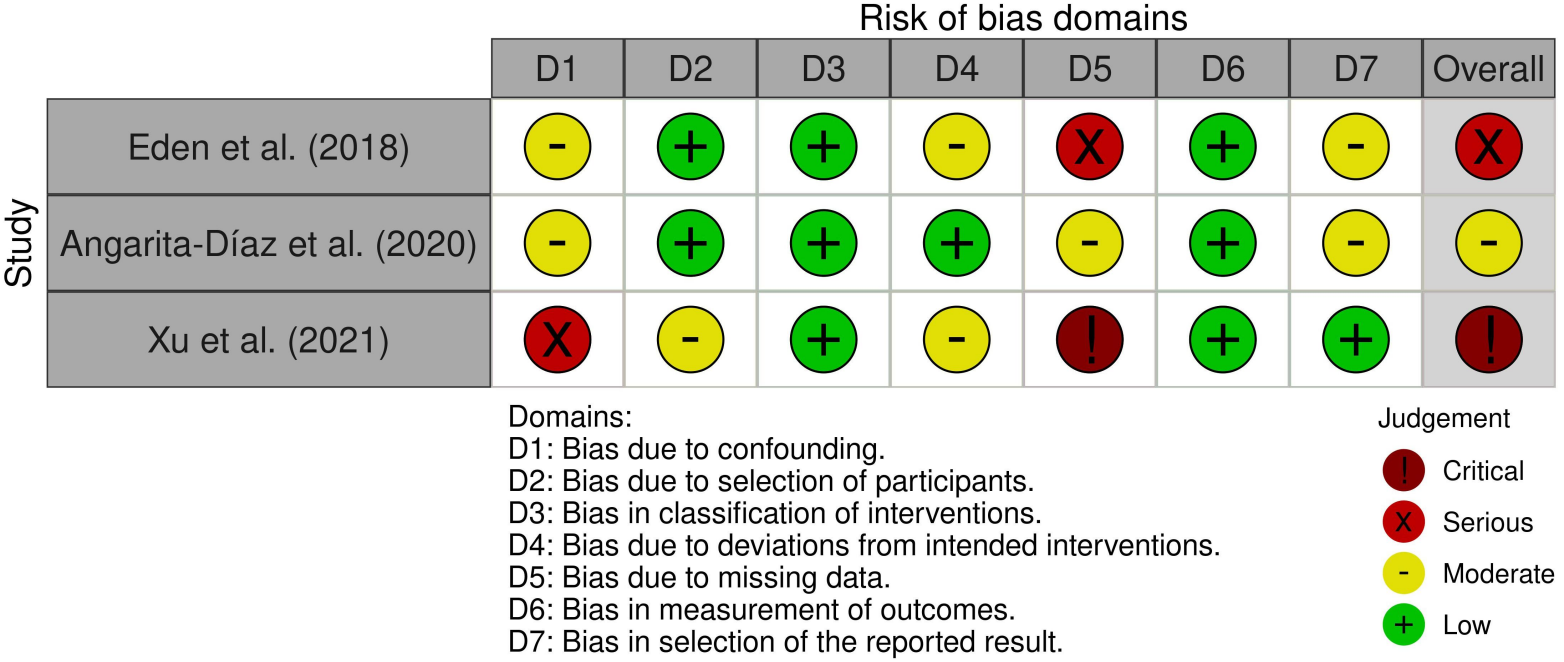
Risk of bias summary of the included non-randomized interventions.

### Quality assessment of *in vitro* studies (QUIN)

The five *in-vitro* studies were appraised using the QUIN tool (21), scored across 12 domains (0–2 each, maximum 24). All five were classified as high quality: Shino et al. (39), Krzyściak et al. (34), Lin et al. (15), and Zeng et al. (37) each scored 18/24 (75%), and Elazim et al. (29) scored 20/24 (83.3%). Despite this classification, all five studies scored zero on the same three domains, namely, sample size calculation, randomization (of sample/specimen allocation), and blinding of outcome assessment. Elazim et al. (29) was the sole exception, scoring full marks on sample size calculation (Table 4).

### Synthesis of results

#### Caries incidence and increment

Of the 13 RCTs, most reported a reduction in caries incidence or increment associated with probiotic intervention. Näse et al. reported a significant reduction in caries risk with *Lactobacillus rhamnosus GG*, most pronounced in children aged 3–4 years (22). Stecksén-Blicks et al. reported a 75% prevented fraction with fluoridated probiotic milk (28). Rodriguez et al. (14), Pahumunto et al. (25), Sandoval et al. (36), and Ibacache et al. (35) similarly reported reduced caries progression or new-lesion development with long-term probiotic-milk consumption. The multi-centre trials reported from Thailand (24, 26, 27) observed that daily or triweekly *L. paracasei SD1* modestly prevented new caries and reversed existing carious lesions. Wongsupa et al. reported significantly fewer caries in the intervention group with the administration of probiotics, though at a greater cost (23).

However, the noted pattern displayed certain heterogeneity. Hasslöf et al. found no significant between-group difference in caries prevalence after 12 months of *Limosilactobacillus reuteri* oral drops, with a high overall rate of recurrent lesions (67%) in both arms, and reported no beneficial effect on plaque or gingival inflammation (10). Villavicencio et al. reported reductions in salivary Lactobacillus counts and improved buffering capacity but did not report a corresponding reduction in caries progression (32).

#### Salivary and plaque microbial counts

Several trials reported reductions in salivary and/or plaque *Streptococcus mutans* counts with probiotic administration (26, 27, 30, 33). The *Lactobacillus* counts were less consistent, with Tehrani et al. (30) reporting no change in *Lactobacillus* despite reduction with *Streptococcus mutans*, whereas Manmontri et al. (26) reported an increase in total salivary lactobacilli persisting at least 6 months post-discontinuation. Eden et al. (31) found no significant change in salivary S. mutans, Lactobacillus, pH, or salivary IgA after 3 weeks of probiotic yogurt, with only salivary buffering capacity showing a significant increase. Angarita-Díaz et al. reported reduced cariogenic microorganism counts with combined *L. rhamnosus GG and B. longum*-supplemented milk (33).

#### Other biomarkers and secondary outcomes

Sandoval et al. reported a reduction in salivary human β-defensin-3 alongside reduced caries occurrence (36). Wattanarat et al. reported enhanced salivary HNP1-3 levels with concurrent S. mutans reduction in children with severe ECC (27). Xu et al. found no difference in salivary microbial α-diversity between groups, though β-diversity (community structure) differed with probiotic yogurt consumption (38).

#### *In-vitro* mechanistic evidence

Across the five *in-vitro* studies (15, 29, 34, 37, 39), *Lactobacillus* consistently demonstrated antimicrobial and antibiofilm activity against *S. mutans.* In two studies, growth inhibition agaisnt *Candida albicans* was evaluated (37, 39). Krzyściak et al. (34) and Lin et al. (15) reported substantial reductions (70%–90%) in biofilm biomass and colony counts using clinically derived multispecies biofilms. Zeng et al. additionally demonstrated reduced extracellular polysaccharide production and virulence-gene expression with *L. plantarum* (37). Shino et al. reported that a commercial probiotic preparation inhibited Candida albicans but was significantly less effective than ketoconazole (39). As mechanistic, non-clinical evidence, these findings support biological plausibility for the clinical trials and non-randomized findings above, but do not independently establish a clinical effect (Table 3).

**Table 3:** Characteristics of included in-vitro studies.

S.No.	Author (year)	Country	Study design	Sample size (I/C)	Intervention administered	Dose quantity	Samples collected	Techniques	Outcomes assessed	Key findings
1	Shino et al. (2016) (39)	India	*In vitro* laboratory study	Oral swabs from children (3–6 years) with ECC; 20 clinical Candida albicans isolates	Vizylac® (lactic acid Bacillus) compared with 0.2% chlorhexidine, coconut oil, and 2% ketoconazole	4 μL/disc on 6 mm filter paper discs; C. albicans suspension standardized to 0.5 McFarland	Oral swabs from buccal, lingual, proximal and cervical tooth surfaces	Sabouraud dextrose agar culture, germ tube test, Dalmau plate culture, Kirby–Bauer disc diffusion assay, inhibition zone measurement	Antifungal activity against Candida albicans (zone of inhibition)	Mean inhibition zones: ketoconazole 22.3 mm, chlorhexidine 21.8 mm, coconut oil 16.8 mm, probiotics 13.5 mm. Probiotics inhibited C. albicans but were significantly less effective than ketoconazole.
2	Krzyściak et al. (2017) (34)	Poland	*In vitro* laboratory study	59 children with ECC; clinical S. mutans and Candida albicans isolates	Ligilactobacillus salivarius (HM6 Paradens)	S. mutans standardized to 1 × 10^6^ CFU/mL; C. albicans to 1 × 10^4^ CFU/mL; 50 μL probiotic added to biofilms	Dental plaque from children with ECC	Mono- and dual-species biofilm model, crystal violet assay, CFU counts, SEM, flow cytometry	Biofilm biomass, CFU counts, biofilm architecture, hyphal formation	Significantly reduced S. mutans and C. albicans biofilm biomass and CFU counts, disrupted extracellular matrix and inhibited hyphal formation.
3	Lin et al. (2017) (15)	China	*In vitro* laboratory study	20 children (3–5 years) with active caries; 12 clinical S. mutans isolates; plaque-derived multispecies biofilms	Lactobacillus casei Shirota, L. casei LC01, L. plantarum ST-III, Lacticaseibacillus paracasei LPC37	Probiotic suspensions standardized to 1.5 × 10^8^ CFU/mL	Saliva and dental plaque from children with active caries	Co-culture inhibition assay, hydroxyapatite multispecies biofilm model, qPCR, colony counting	Growth inhibition, biofilm composition, bacterial counts	All four probiotics inhibited approximately 70%–90% of S. mutans isolates and significantly reduced cariogenic bacterial counts within multispecies biofilms.
4	Elazim et al. (2022) (29)	Egypt	*In vitro* laboratory study (embedded within an observational study)	7 breast milk, 7 probiotic formula, 7 plain formula samples; bacterial isolates from infant saliva	Probiotic-supplemented infant formula (Nan® 1 Optipro; Bifidus culture) compared with breast milk, plain formula and nutrient broth	0.5 mL milk/formula inoculated with 0.5 McFarland bacterial suspension	Breast milk, probiotic infant formula, plain formula; salivary S. mutans and Lactobacillus isolates	Anaerobic culture on selective media; CFU enumeration	Growth of S. mutans and Lactobacillus	Breast milk showed the lowest S. mutans counts, followed by probiotic formula; plain formula showed the highest bacterial growth (*p* < 0.001).
5	Zeng et al. (2023) (37)	USA	*In vitro* laboratory study	Clinical isolates from 10 children with severe ECC; biofilm experiments using isolates from 3 children	Lactobacillus plantarum ATCC 14,917 (selected after screening three probiotic strains); plantaricin also evaluated	L. plantarum added to planktonic cultures and biofilms; plantaricin tested at 0–400 μg/mL	Clinical Streptococcus mutans and Candida albicans isolates from children with severe ECC	Multispecies planktonic assay, biofilm model, CFU counts, CLSM, EPS quantification, qRT-PCR, MIC assay	Growth inhibition, biofilm biomass, EPS production, virulence gene expression, antimicrobial activity	L. plantarum significantly reduced planktonic growth, biofilm biomass, EPS production and virulence gene expression of S. mutans and C. albicans. Plantaricin demonstrated antimicrobial activity.

**Table 4:** Quality assessment of in-vitro studies using the QUIN tool.

S.No.	Study	Aim	Sample	Sample size calculation	Methodology	Control	Randomization	Standardization	Blinding	Statistics	Outcomes	Conclusion	COI/Funding	Total	%	Quality
1	Shino et al. (39)	2	2	0	2	2	0	2	0	2	2	2	2	18	75	High
2	Krzyściak et al. (34)	2	2	0	2	2	0	2	0	2	2	2	2	18	75	High
3	Lin et al. (15)	2	2	0	2	2	0	2	0	2	2	2	2	18	75	High
4	Elazim et al. (29)	2	2	2	2	2	0	2	0	2	2	2	2	20	83.3	High
5	Zeng et al. (37)	2	2	0	2	2	0	2	0	2	2	2	2	18	75	High

### Publication bias assessment

Because pooling was not undertaken for the review's primary narrative synthesis, publication bias was formally assessed within a supplementary quantitative model restricted to the 8 studies reporting in a form amenable to pooling (14, 23–25, 27, 28, 32, 36).

Several statistical tests were conducted to evaluate the presence of publication bias. The fail-safe *N* was calculated using Rosenthal's approach, yielding a value of *N* = 18, *p* = 0.002. This indicates that 18 additional studies with null results would be required to render the overall findings nonsignificant, suggesting a relatively robust effect. Begg and Mazumdar rank correlation test showed a correlation coefficient of *r* = −0.071, *p* = 0.905 with no significant evidence for publication bias based on rank correlation. Also, Egger's regression test produced an intercept of 0.222, *p* = 0.824, which is not indicative of asymmetry in the funnel plot. Finally, the trim-and-fill procedure estimated that one study might be missing from the analysis. However, the adjusted effect size remained consistent with the observed results, implying that the overall conclusions are unlikely to be substantially influenced by publication bias.

Visual inspection of the funnel plot revealed a generally symmetric distribution of studies around the mean effect size, with most points falling within the expected funnel boundaries. Although one imputed study was suggested by the trim-and-fill method, the overall pattern does not indicate substantial asymmetry (Figure 4).

**Figure 4:**
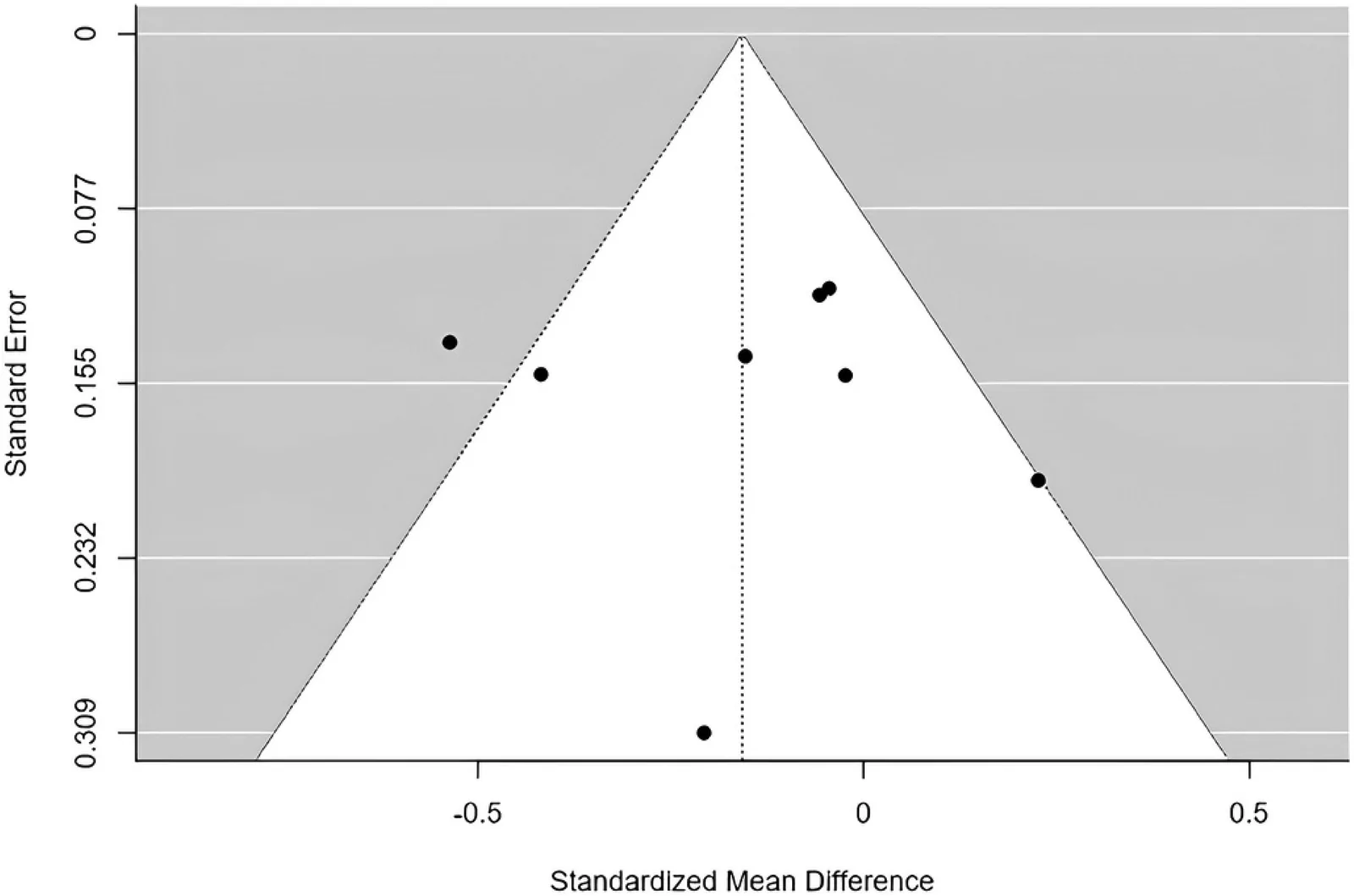
Funnel plot depicting evaluation of publication bias.

### Certainty of evidence (GRADE)

The certainty of evidence was assessed using the Grading of Recommendations Assessment, Development and Evaluation (GRADE) framework across four evidence tiers (40). The overall summary is represented in Table 5. The randomized controlled trials (RCTs), comprising 13 studies with 1,748 participants in the treatment group and 1,746 in the placebo or control group, were graded as moderate certainty. Although RCTs typically begin at high certainty, these studies were downgraded due to a serious risk of bias. However, inconsistency, indirectness, imprecision, and publication bias were judged not serious, thereby maintaining a moderate level of confidence in the effect estimates. The two non-randomized intervention studies, involving 69 participants, were graded as low certainty. These studies start at low certainty because of their design limitations. Additional downgrading was applied for serious risk of bias and imprecision, particularly due to small sample sizes, which limited the reliability of the findings. Other domains were not considered serious, so the overall rating remained low. The single observational study, with 27 participants, was also graded as low certainty. Observational studies begin at low certainty, and this study was further downgraded for serious risk of bias and imprecision. Although no major inconsistency, indirectness or publication bias was present, the small sample size and methodological issues precluded any upgrade and the certainty of evidence was thus low. Finally, the five in-vitro studies, encompassing 130 experimental samples, were graded as very low certainty. *In vitro* evidence is inherently indirect, as it does not directly represent clinical outcomes. These studies were further downgraded for serious risk of bias, inconsistency, indirectness and imprecision. Publication bias was judged not serious, but the overall certainty remained very low, reflecting the limited confidence in translating these findings to clinical practice.

**Table 5:** GRADE framework.

Study design	No. of participants	Risk of bias	Inconsistency	Indirectness	Imprecision	Publication bias	Overall certainty of evidence
Randomized controlled trials-14 studies	Treatment group: 1,748	Serious risk	Not serious	Not serious	Not serious	Not serious	⊕⊕⊕Ο
	Placebo or control group: 1,746						
Non-randomized intervention studies- 2 studies	69	Serious risk	Not serious	Not serious	Serious risk	Not serious	⊕⊕ΟΟ
Observational studies- 1 study	27	Serious risk	Not serious	Not serious	Serious risk	Not serious	⊕⊕ΟΟ
*In-vitro* studies- 5 studies	130	Serious risk	Serious risk	Serious risk	Serious risk	Not serious	⊕ΟΟΟ

Certainty of evidence was assessed using the GRADE framework. ⊕⊕⊕Ο, Moderate; ⊕⊕ΟΟ, low; ⊕ΟΟΟ, very low.

## Discussion

This systematic review synthesized evidence from 13 randomized controlled trials, 3 non-randomized intervention studies, and 5 *in-vitro* studies assessing probiotic interventions on caries incidence, oral microbial counts, and related oral health outcomes in children under 6 years. Overall, the clinical evidence suggests that probiotic supplementation may have a favourable effect on oral health outcomes, with a majority of the studies reporting reductions in salivary and/or plaque *S. mutans* counts and caries incidence. However, these benefits were not consistently observed across all studies. While several trials demonstrated improvements in clinical and microbiological outcomes following probiotic supplementation (14, 22, 28, 35), others reported no significant reduction in caries prevalence or only modest microbiological changes (10, 31, 32), indicating that the available evidence remains heterogeneous.

A notable finding across the included studies was the strain-specific nature of probiotic efficacy. Trials evaluating *Lactobacillus paracasei* SD1 consistently demonstrated reductions in *S. mutans* counts, delayed caries progression, and improvements in host antimicrobial peptides following long-term milk supplementation (24–27). A similar result was obtained with studies using *Lactobacillus rhamnosus* SP1 wherein a favourable effect on caries progression was reported (14, 22, 28, 30, 31). Another strain examined in the included studies is *Limosilactobacillus reuteri*, however, no significant changes were reported with this particular strain (10). Two of the included studies considered a combination of probiotic strains and reported a notable effect on salivary and plaque microbial counts, potentially implying that a synergistic effect may also be observed with the use of probiotic strains (33, 38). Collectively, these findings suggest that probiotic efficacy should be considered strain-specific rather than a uniform class effect.

The direction of the microbial-count findings identified in the present review is broadly consistent with Meng et al's meta-analysis reporting reduced salivary *S. mutans* counts with probiotic use, particularly with *Lactobacillus rhamnosus* strains (9). Similar reports were identified in the clinical trial reported by Staszczyk et al. wherein chewing tablets coated with *Lactobacillus* strains demonstrated a notable reduction in caries progression at the end of 12 months (12). While the impact of *Limosilactobacillus reuteri* strain seemed to have minimal effect, its administration to the mothers during the gestational period has been reported to be of greater efficacy (41). These findings require interpretation with caution due to the varied nature of the included population and the lack of standardization of the administered probiotic intervention.

The present review extends previous evidence by incorporating recent randomized trials, non-randomized studies, and mechanistic *in vitro* investigations, thereby providing a broader evaluation of probiotic interventions than earlier reviews focused primarily on microbiological outcomes. Furthermore, unlike previous systematic reviews that evaluated probiotics in children across wider age ranges (42, 43), the present review specifically focused on infants and preschool children, the population at greatest risk of developing early childhood caries.

### Biological plausibility and mechanistic evidence

The five *in-vitro* studies provide consistent mechanistic support for the antimicrobial and antibiofilm activity of Lactobacillus species against *S. mutans* and, in two studies, *Candida albicans* (15, 29, 34, 37, 39). Probiotic exposure reduced bacterial growth, disrupted extracellular polymeric substance formation, inhibited biofilm maturation, decreased virulence gene expression, and suppressed hyphal formation in dual-species biofilm models. This offers biological plausibility for the clinical and microbiological findings above, but the mechanistic evidence should not be read as clinical confirmation. This is attributed to the fact that an *in-vitro* model cannot accurately replicate the oral cavity's ecological complexity, salivary flow, or host immune interaction. This is another reason as to why this tier was downgraded for indirectness rather than treated as directly supportive clinical evidence during quality assessment.

The present review's rationale was centered on the premise of the milk–oral–gut axis, that these interventions act by modulating the gut microbiome, which in turn influences oral microbial ecology. Although the included interventions consisted of orally administered probiotics, none of the included clinical studies directly evaluated gut microbiome composition or function. Therefore, this review cannot establish whether the observed oral health benefits were mediated through alterations in the gut microbiome. The available evidence instead supports the possibility that probiotics improve oral health through local oral mechanisms, including competitive inhibition of cariogenic bacteria, biofilm disruption, production of antimicrobial metabolites, and modulation of host immune responses. Future studies incorporating simultaneous assessment of oral and gut microbiomes are required to determine whether gut-mediated pathways contribute to these clinical effects.

The presented evidence base highlights the efficacy of specific strains of bacteria and dietary interventions which have provided simulated and live changes in the oral health outcomes. These findings emphasize the definitive association between modulations of gut health and oral health. Several mechanisms independent of gut modulation may also explain the observed clinical findings. These include competitive inhibition of cariogenic microorganisms, production of antimicrobial compounds such as bacteriocins, interference with biofilm formation, competition for adhesion sites, and modulation of local innate immune responses, including increased antimicrobial peptide expression. These mechanisms are supported by both the clinical and laboratory evidence included in this review. Future trials pairing simultaneous gut and oral microbiome sampling within the same cohort would be needed to determine whether changes in gut microbial composition contribute to improvements in oral health or whether the observed effects are primarily mediated through local oral mechanisms.

The GRADE assessment further contextualizes the findings of this review by demonstrating that confidence in the available evidence varies across study designs. The overall certainty of evidence ranged from moderate to low and very low certainty based on the study design and conduct, suggesting that the findings of this review be interpreted with appropriate caution. Further high-quality, adequately powered randomized controlled trials employing standardized interventions and outcome measures are required to strengthen the certainty of the evidence and inform clinical recommendations.

### Strengths and limitations

The present review has several strengths. A blinded screening protocol in accordance with PRISMA guidelines was conducted to ensure unbiased and independent study selection. A pre- piloted protocol and data extraction form was developed to prevent further hurdles during the conduct of the review. Appropriate quality assessment tools were utilized for the evidence assessment. Publication bias was analyzed and GRADE was further assessed to explore the quality of the evidence presented. Additionally, integrating clinical and mechanistic evidence allowed a more comprehensive understanding of probiotic interventions than would have been possible by evaluating clinical studies alone.

Several limitations should also be acknowledged. Considerable clinical and methodological heterogeneity existed across the included studies with respect to probiotic strain, dosage, delivery vehicle, intervention duration, baseline caries risk, and outcome assessment, limiting direct comparison between studies. Although the review was designed to evaluate a broad range of microbiome-modulating interventions, the final body of eligible evidence consisted almost exclusively of probiotic-based interventions, with no eligible clinical studies evaluating synbiotics, prebiotics, or other dietary microbiome-modulating strategies. Finally, none of the included studies evaluated alterations in the gut microbiome, precluding conclusions regarding the proposed oral–gut microbiome relationship.

### Implications for clinical practice and future research

Considering the available evidence, probiotic supplementation may represent a promising adjunctive strategy for improving oral microbial profiles and reducing caries progression in young children. However, the findings of this review are not sufficient to deliver a definitive conclusion owing to the heterogeneity of the reports and the varying study methodology and quality. Rather, probiotics should be considered a potential adjunct to established preventive measures, including fluoride exposure, dietary counselling, and appropriate oral hygiene practices.

Future research should prioritise adequately powered multicentre randomized controlled trials using standardized probiotic strains, doses, delivery vehicles, intervention durations, and caries outcome measures to improve comparability across studies. Long-term follow-up is required to determine the sustainability of probiotic effects on caries incidence. Additionally, future studies should incorporate simultaneous assessment of oral and gut microbiomes to clarify the biological pathways underlying probiotic action and determine whether systemic gut-mediated mechanisms contribute to improvements in oral health.

## Conclusion

Probiotic supplementation as dietary interventions or adjunct to dietary supplementation show promise in improving the oral microbial profile and reducing caries progression in children aged less than 6 years. The findings further suggest that probiotic efficacy is likely strain-specific and the biological plausibility of individual strains cannot be definitively determined without further high-quality, well-designed trials evaluating the same. Furthermore, simultaneous evaluation of oral and gut microbiome compositions is essential to clearly understand the link between modulation of the gut microbiome and its effect on the oral microbiome.

## Data Availability

The original contributions presented in the study are included in the article/supplementary material, further inquiries can be directed to the corresponding author..
